# Genomic Analysis of Halotolerant Bacterial Strains *Martelella soudanensis* NC18^T^ and NC20

**DOI:** 10.4014/jmb.2208.08011

**Published:** 2022-10-13

**Authors:** Jung-Yun Lee, Dong-Hun Kim

**Affiliations:** 1 Groundwater Environment Research Center, Korea Institute of Geoscience and Mineral Resources, Daejeon 34132, Republic of Korea; 2 Department of Biological Science and Biotechnology, Microbiology and Biotechnology, Chungbuk National University, Cheongju 28644, Republic of Korea

**Keywords:** *Martelella soudanensis*, whole-genome sequencing, functional categorization, salt tolerance

## Abstract

Two novel, halotolerant strains of *Martelella soudanensis*, NC18^T^ and NC20, were isolated from deep subsurface sediment, deeply sequenced, and comparatively analyzed with related strains. Based on a phylogenetic analysis using 16S rRNA gene sequences, the two strains grouped with members of the genus *Martelella*. Here, we sequenced the complete genomes of NC18^T^ and NC20 to understand the mechanisms of their halotolerance. The genome sizes and G+C content of the strains were 6.1 Mb and 61.8 mol%, respectively. Moreover, NC18^T^ and NC20 were predicted to contain 5,849 and 5,830 genes, and 5,502 and 5,585 protein-coding genes, respectively. Both strains contain the identically predicted 6 rRNAs and 48 tRNAs. The harboring of halotolerant-associated genes revealed that strains NC18^T^ and NC20 might tolerate high salinity through the accumulation of potassium ions in a “salt-in” strategy induced by K^+^ uptake protein (kup) and the K^+^ transport system (trkAH and kdpFABC). These two strains also use the ectoine transport system (dctPQM), the glycine betaine transport system (proVWX), and glycine betaine uptake protein (opu) to accumulate “compatible solutes,” such as ectoine and glycine betaine, to protect cells from salt stress. This study reveals the halotolerance mechanism of strains NC18^T^ and NC20 in high salt environments and suggests potential applications for these halotolerant and halophilic strains in environmental biotechnology.

## Introduction

Since the genus *Martelella* was first proposed by Rivas *et al*. [1], several species have been isolated worldwide from a variety of environments, including saline lakes [1], the roots or mud plate of halophytes [2-6], soil from mangrove roots [3, 7], and saline soil with petroleum contamination [8]. These species are highly halotolerant (up to 11% salinity) and are mostly associated with marine environments [1-8].

Halophiles and halotolerant bacteria are able to grow in the absence and presence of high salt concentrations [9]. In recent decades, a large number of halophilic bacteria have been isolated and taxonomically characterized, and many of them are part of the phyla Proteobacteria, Cyanobacteria, Firmicutes, Actinobacteria, Spirochaetes, and Bacteroidetes [10]. To survive the osmotic stress caused by high salt concentrations, microorganisms employ two main strategies: "salt-in" and "compatible solute" [11]. In the salt-in strategy, salts (mainly K^+^) are accumulated in the cytoplasm to compete with the external high concentrations of Na^+^ and maintain the osmotic intracellular pressure balance [12]. It requires osmotic adaptation of the intracellular enzymatic machinery to the presence of salt so that the proteins can maintain a suitable structure and activity at high salt concentrations [13]. In the compatible solute strategy, small organic molecules, commonly ectoine and glycine betaine, are accumulated for adaptation to considerable osmotic stress in halophiles and to synthesize and transport compatible solutes [10, 14, 15].

*Martelella soudanensis* NC18^T^ and NC20 were isolated from a sediment sample collected from borehole effluent originating 714 m below the subsurface at the Soudan Iron Mine in northern Minnesota, USA. Based on the results of phylogenetic and genomic analyses, these two isolates clearly formed a phylogenic lineage with members of the genus *Martelella* [16]. However, these strains could also be clearly distinguished from other closely related genera based on their ability to grow at higher salt concentration. Therefore, we propose that strains NC18^T^ and NC20 represent a novel species of a genus, and thus we have named them *Martelella soudanensis*. To understand the mechanisms of the halotolerance of *M. soudanensis* strains NC18^T^ and NC20, the complete genomes of both strains were sequenced and predicted for halotolerance-associated genes. This study provides a theoretical basis for the halophilic characteristics of the genus *Martelella* and suggests potential applications for these halotolerant and halophilic strains in environmental biotechnology.

## Materials and Methods

### Whole-Genome Sequencing, Assembly, and Annotation

The halophiles *M. soudanensis* NC18^T^ and NC20 were isolated from Soudan Underground Mine State Park in Soudan, Minnesota, USA [16]. Genomic DNA was extracted and purified using an AccuPrep Genomic DNA Extraction Kit (Bioneer, Korea) according to the manufacturer’s instructions.

The complete genomes of strains NC18^T^ and NC20 were sequenced by combining Illumina MiSeq and PacBio RSII high-throughput sequencing technology at CJBioscience, Inc. (Korea). The raw sequences from MiSeq were assembled using the SPAdes assembler 3.9.0 (http://cab.spbu.ru/software/spades/), and quality trimming was performed using Trimmomatic 0.36 [17]. Assembled sequences were cleaned from PhiX sequences with BBMap 38.32 [18]. Whole-genome sequencing was performed using PacBio SMRT Link 7.0.1 with HGAP4 protocol (Pacific Biosciences, USA). Hybrid genome assembly was generated using the program Pilon (version 1.22) and reassembled using quality control MiSeq data. Meanwhile, contigs were assembled from PacBio data. The error-corrected assembly was tested for possible circularity using Circlator v1.4.0 [19]. The whole genome sequence was checked for contamination using ContEst16 [20].

The whole genomes of strains NC18^T^ and NC20 were annotated by the EzBioCloud database. Protein coding sequences (CDSs) were performed by Prodigal v.2.6.2 [21]. The tRNAscan SE 1.3.1 [22] and Rfam 12.0 databases [23] were used to predict transfer RNA (tRNA) genes and ribosomal RNA (rRNA) genes, respectively. CRISPR repeats were identified by PilerCR 1.06 [24] and the CRISPR recognition tool (CRT) 1.2 [25]. Graphical circular genome maps of the genomes of the two strains were generated using the EzBioCloud Comparative Genomics Database (www.ezbiocloud.net/genome). Gene prediction and functional annotation were based on the KEGG database [26] and the clusters of orthologous groups (COG) database [27] were performed using EggNOG 4.5 (http://eggnogdb.embl.de). To obtain detailed functional annotation, the predicted CDSs were compared with SEED [28] databases using the RAST server [29].

### Phylogenetic and Phylogenomic Analysis

The 16S rRNA gene sequence similarity of the two strains and closely related taxa was compared using the EzBioCloud server (www.ezbiocloud.net) [30]. The 16S rRNA gene sequences were aligned using the CLUSTAL X software program [31], and gaps were edited in the BioEdit program [32]. The phylogenetic trees were constructed using the MEGA 6.0 software with neighbor-joining, maximum-likelihood, and maximum-parsimony methods [33]. Statistical reliability was assessed from 1,000 bootstrap replicates. The G+C content of the genomic DNA was determined from each genome sequence. The average nucleotide identity (ANI) and in silico DNA-DNA hybridization (DDH) values were calculated by using the EZGenome web service (www.ezbiocloud.net/tools/ani) and Genome-to-Genome Distance Calculator (http://ggdc.dsmz.de/ggdc.php), respectively [34, 35]. Gene clusters were analyzed with publicly available genomic sequences of *Martelella mediterranea* DSM 17316^T^ (GCF_002043005), *Martelella endophytica* YC6887^T^ (GCF_000960975), and *Martelella lutilitoris* GH2-6^T^ (GCF_005924265) using Mauve (version 20150226) to understand the salt tolerance mechanism [36].

### Availability of Data and Materials

Strains NC18^T^ and NC20 were deposited in the Korean Collection for Type Culture (KCTC) and NITE Biological Resource Center (NBRC) under the deposit numbers KCTC 82174^T^=NBRC 114661^T^ and KCTC 82175=NBRC 114662, respectively. The 16S rRNA gene sequences of strains NC18^T^ and NC20 were deposited in GenBank/EMBL/DDBJ under accession numbers MT367774 and MT367775, respectively. The genomic sequences of strains NC18^T^ and NC20 were deposited at DDBJ/ENA/GenBank under accession numbers CP054858-CP054860 and CP054861-CP054863, respectively.

## Results and Discussion

### Phylogenetic and Phylogenomic Analysis

**Fig. 1:**
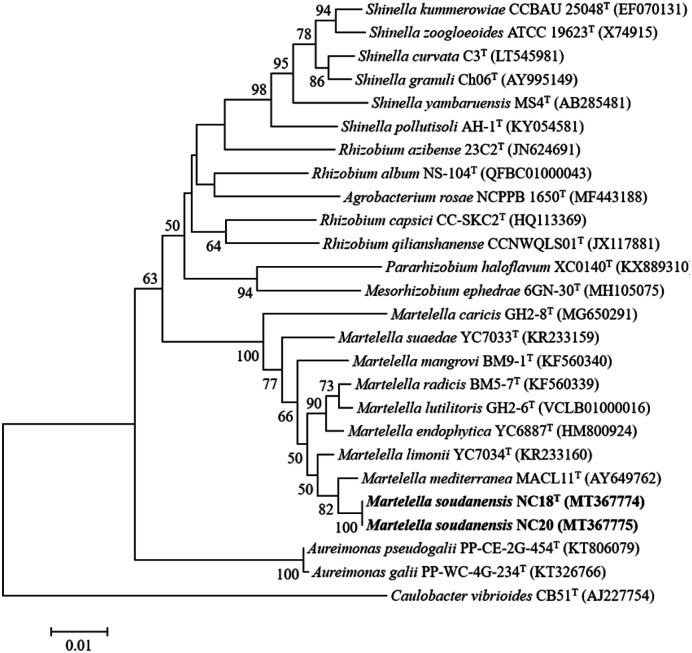
Phylogenetic tree based on 16S rRNA gene sequences of *Martelella soudanensis* NC18^T^ and NC20 with other related taxa. The tree was constructed using the Jukes–Cantor model and neighbor-joining method with 1,000 bootstrap replications [16]. Bootstrap values above 50% are shown next to the branches. Bar, 0.01 nucleotide substitution per position.

The 16S rRNA gene sequences of *M. soudanensis* NC18^T^ and NC20 showed 100% similarity. Comparative 16S rRNA gene sequence analyses revealed that *M. soudanensis* NC18^T^ and NC20 were most closely related to *M. mediterranea* DSM 17316^T^ (99.0%), *Martelella limonii* YC7034^T^ (98.6%), *M. endophytica* YC6887^T^ (98.1%), *Martelella mangrovi* BM9-1^T^ (97.9%), *M. lutilitoris* GH2-6^T^ (97.9%), *Martelella radicis* BM5-7^T^ (97.6%), *Martelella suaedae* YC7033^T^ (97.6%), and *M. caricis* GH2-8^T^ (97.2%) [16]. The neighbor-joining, maximum-likelihood, and maximum-parsimony phylogenetic analyses revealed that the two isolates formed a lineage within the clade of the genus *Martelella* but are separate from the clade constituted of the species *M. mediterranea* and *M. limonii* (Figs. 1, S1). The ANI and dDDH values between strains NC18^T^ and NC20 were 99.9% and 100%, respectively [16]. These results revealed that the two strains belonged to a single species. On the other hand, the ANI and DDH values between *M. soudanensis* NC18^T^ and *M. mediterranea* DSM 17316^T^ were determined to be 88.1% and 34.9%, respectively. The ANI and DDH values were found to be higher than the threshold values proposed to distinguish two different species (ANI 95% and DDH 70%) [37, 38].

### General Genomic Characteristics and Annotation

The general genomic features of *M. soudanensis* NC18^T^ and NC20 are listed in Table 1. The complete genome sequence of strain NC18^T^ comprised a circular chromosome of 6,109,459 bp containing 5,531 functional CDSs, 292 pseudogenes, 6 rRNAs, and 48 tRNAs with an average G+C content of 61.8%. Additionally, the genome of strain NC20 comprised 6,109,677 bp and had a G+C content of 61.8%. The genome contained 5,467 functional CDSs, 275 pseudogenes, 6 rRNA, and 48 tRNA genes (Fig. S2).

### Functional Categorization

**Fig. 2:**
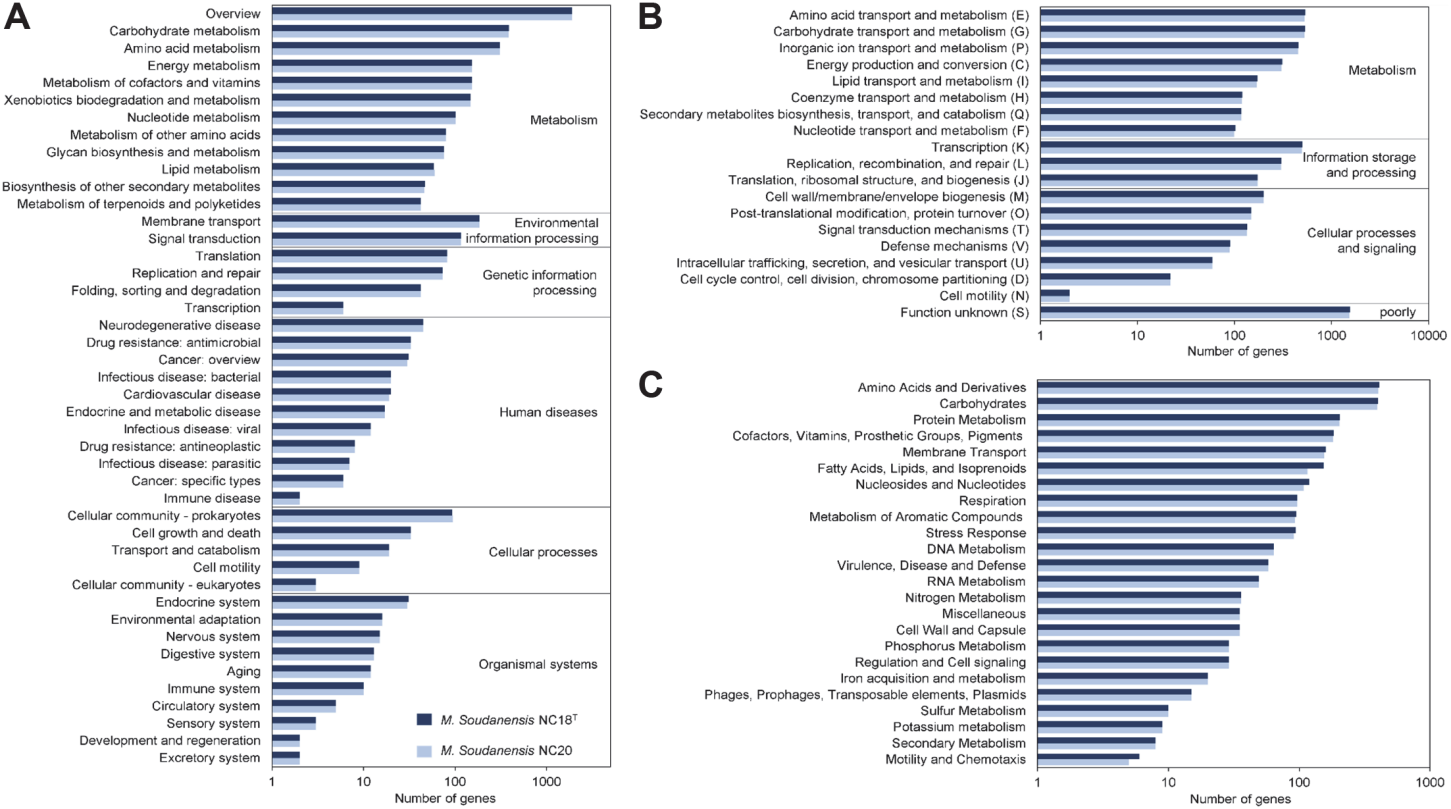
Functional distribution of the *Martelella soudanensis* NC18^T^ and NC20 genomes. The x-axis represents the number of genes annotated, and the y-axis stands for the functional distribution. **A**. KEGG annotation. **B**. COG annotation. **C**. SEED annotation.

The functionally encoded genetic features in *M. soudanensis* NC18^T^ and NC20 were categorized according to the KEGG, COG, and SEED databases. The 5,823 CDSs for *M. soudanensis* NC18^T^ and 5,742 CDSs for *M. soudanensis* NC20 were assigned to 4,424 and 4,413 KEGG identifiers (Table S1), 3,984 and 3,937 COG identifiers (except for function unknown) (Table S2), and 2,324 and 2,248 SEED identifiers (Table S3), respectively. In the KEGG analysis, carbohydrate and amino acid metabolism were mainly abundant, indicating that *M. soudanensis* is capable of using a variety of carbon sources (Fig. 2A, Table S1) [16]. In particular, genes related to membrane transport and signal transduction were ranked high, suggesting a higher tolerance level of *Martelella* under harsh salt conditions. In the COG analysis, the genes for amino acid transport and metabolism (E), carbohydrate transport and metabolism (G), inorganic ion transport and metabolism (P), and transcription (K) were highly identified except for function unknown (S) (Fig. 2B, Table S2). These categories are closely linked to the nutrients obtained from various environments and the maintenance of survival [39]. Function unknown (S) accounted for a large portion, indicating the current lack of understanding of *M. soudanensis* genomes.

In the SEED analysis, the most abundant functions were associated with the amino acid and derivatives, carbohydrate, protein metabolism, cofactors, vitamins, prosthetic groups, pigments, and membrane transport subsystems (Fig. 2C, Table S3). Overall, the functional gene categories in the KEGG, COG, and SEED profiles for *M. soudanensis* NC18^T^ and NC20 were classified similarly.

### Salt Tolerance of *M. soudanensis* NC18^T^ and NC20

Osmotic adaptation is essential for bacterial survival in a high salt environment. If the osmotic pressure of the environment is higher than that of the cells, water outflow occurs, resulting in dehydration. Thus, cells maintain homeostasis by reaching an osmotic balance through a process called osmotic regulation. As a primary response, preservation of cell osmotic pressure involves water efflux [40] and accumulation of potassium (K^+^) for water retention [41].

**Fig. 3:**
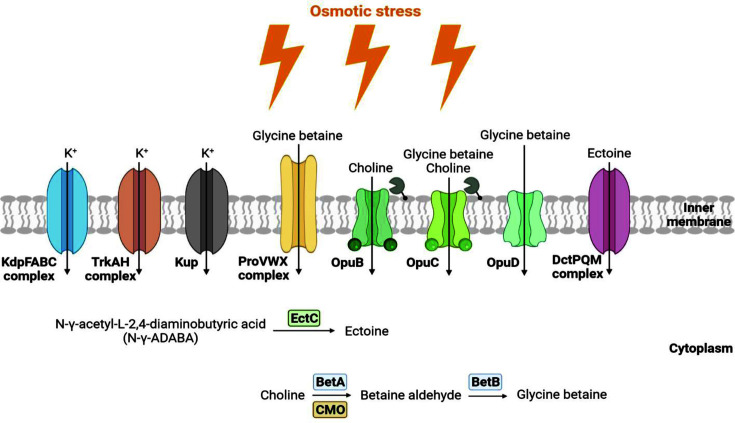
Schematic representation of the salt tolerance mechanisms in *Martelella soudanensis* NC18^T^ and NC20 based on genome analyses. Genomes of strains NC18^T^ and NC20 deploy two main strategies to increase the salt tolerance: "salt-in" and "compatible solute". The figure was created using the BioRender (http://biorender.com).

After this primary response, osmoprotectants that are more efficient than K^+^, such as glycine betaine and ectoine, accumulate. These osmoprotectants, as compatible solutes, are either biosynthesized or salvaged from the environment [42-44]. The annotation results of the *M. soudanensis* NC18^T^ and NC20 genomes revealed that some homologous proteins related to halotolerance-associated genes showed two main strategies: "salt-in" and "compatible solute" (Table S4 and Table S5) [11]. *M. soudanensis* NC18^T^ and NC20 use the ectoine transport system (DctPQM), the glycine betaine transport system (ProVWX), ectoine transporter (YiaN), and glycine betaine or/and choline selective transporters (OpuB, OpuC, OpuD, and *TC.BCT*) to accumulate “compatible solutes”, such as ectoine and glycine betaine, to protect cells from salt stress (Fig. 3) [45-49]. After uptake, choline is converted into glycine betaine by a family of oxidoreductases, such as BetA, BetB, and *CMO* [41, 50, 51]. In addition, L-ectoine synthase (EctC), a key enzyme in the production of ectoine, was also identified [52]. Another compatible solute, Nε-acetyl-ß-lysine, is unique to methanogenic archaea and protects the cell walls against salt stress. A gene potentially encoding lysine-2,3-aminomutase (KamA), which is assumed to catalyze Nε-acetyl-ß-lysine formation from alpha-lysine, which is commonly found in methanogenic archaea, has also been identified. Previous studies have suggested that horizontal gene transfer may occur within bacteria and methanogenic archaea by comparing the phylogenetic relationships between lysine 2,3-aminomutase-coding genes and 16S RNA genes [53]. The oxidoreductase PepQ was presumed to protect against damage caused by increasing salt concentrations in cells [54]. These two strains also have a K^+^ uptake system (TrkAH), K^+^ uptake protein (Kup), and K^+^ transport system (KdpFABC) used in the “salt-in” strategy, which can perform one-way transport of K^+^ into the cytoplasm and maintain osmotic pressure to increase salt resistance (Fig. 3) [41, 55, 56]. The compatible solutes also have protection, stabilization and catalysis functions, which make them useful for industrial applications, such as cosmetics, health care, and biotechnology [54].

### Comparative Analyses of Halotolerant-Associated Gene Clusters

**Fig. 4:**
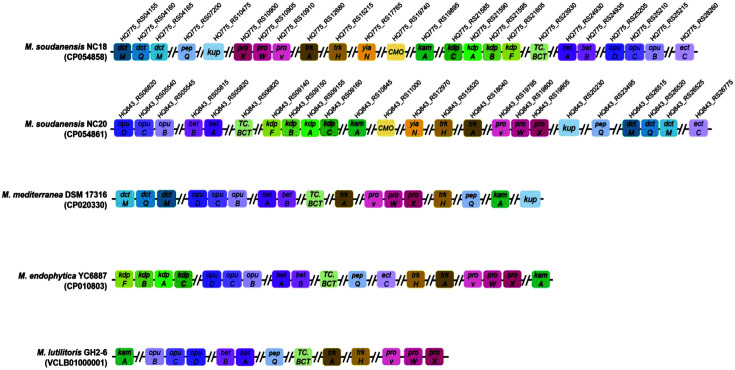
Gene cluster organization and comparison of the halotolerant-associated genes identified in the *Martelella* genomes. From top to bottom, the genomes of strains NC18^T^, NC20, DSM 17316^T^, YC6887^T^, and GH2-6^T^ are shown. Genes are colored according to their functional annotations. Italicized letters indicate locus tags of strains NC18^T^ and NC20, respectively.

The halotolerant-associated gene clusters present in the genomes of *M. soudanensis* NC18^T^, *M. soudanensis* NC20, *M. mediterranea* DSM 17316^T^, *M. endophytica* YC6887^T^, and *M. lutilitoris* GH2-6^T^ were compared using Mauve (Fig. 4, Table 2). A comparison of the gene clusters involved in the salt-in strategy shows that all five strains had *trkA* and *trkH* genes encoding K^+^ uptake proteins while *M. soudanensis* NC18^T^, *M. soudanensis* NC20, and *M. mediterranea* DSM 17316^T^ contained additional *kup* genes encoding K^+^ uptake proteins. Moreover, *M. soudanensis* NC18^T^, *M. soudanensis* NC20, and *M. endophytica* YC6887^T^ had *kdpF*, *kdpA*, *kdpB*, and *kdpC* genes related to the K^+^ transport system. Comparing the gene clusters related to the compatible solute strategy, all five strains had *betA*, *betB*, *pepQ*, *kamA*, *proV*, *proW*, *proX*, *TC.BCT*, *opuB*, *opuC*, and *opuD* genes related to transport and conversion of glycine betaine. Furthermore, *M. soudanensis* NC18^T^, *M. soudanensis* NC20, and *M. mediterranea* DSM 17316^T^ had *dctP*, *dctQ*, and *dctM* genes encoding the ectoine transport system. *M. soudanensis* NC18^T^, *M. soudanensis* NC20, and *M. endophytica* YC6887^T^ had an *ectC* gene encoding L-ectoine synthase. In particular, the *CMO* gene related to glycine betaine conversion and the *yiaN* gene, an ectoine transporter, were found only in *M. soudanensis* NC18^T^ and *M. soudanensis* NC20. The previously reported NaCl%concentrations for the growth of *M. mediterranea* DSM 17316^T^, *M. endophytica* YC6887^T^, and *M. lutilitoris* GH2-6^T^ ranged from 0.0~5.0, 0.0~9.0, and 0.5~9.0, respectively [1, 2, 6], whereas those for *M. soudanensis* NC18^T^ and *M. soudanensis* NC20 ranged from 0.0~13.0 [16]. In terms of the halotolerance mechanism, *M. soudanensis* NC18^T^ and *M. soudanensis* NC20 have more genes involved in K^+^ uptake and transport for the salt-in strategy and additional genes involved in ectoine transport and synthesis for the compatible solute strategy.

Consequently, *M. soudanensis* NC18^T^ and *M. soudanensis* NC20 have more diverse halotolerant-associated gene clusters that support the maintenance of a normal metabolic capacity under high salinity conditions. With the metabolic diversity, low nutritional requirements, and genetic mechanisms of adaptation to harsh conditions such as high ionic strength, halophiles are considered potential unique natural sources for the discovery of bioactive compounds and compatible solutes including novel and/or extraordinary enzymes [57, 58]. These biomolecules are valuable and show commercial potential in the food, pharmaceutical, biomedical, industrial, and environmental fields [59, 60, 61]. Therefore, our results should provide new insights into the halotolerance mechanism of halotolerant and halophilic microbes and their potential applications in environmental biotechnologies.

## Supplemental Materials

Supplementary data for this paper are available on-line only at http://jmb.or.kr.

## Figures and Tables

**Table 1:** Genomic features of *Martelella soudanensis* NC18^T^ and NC20.

Feature	*M. soudanensis* NC18^T^	*M. soudanensis* NC20
Genome size (bp)	6,109,459	6,109,677
G+C content (%)	61.8	61.8
Total genes	5,877	5,796
Total number of CDSs	5,823	5,742
Functional CDSs	5,531	5,467
Pseudogenes	292	275
rRNAs	6	6
tRNAs	48	48

**Table 2:** *Martelella soudanensis* NC18^T^- and NC20-specific genes related to salt tolerance based on the KEGG database.

Salt tolerance: Compatible solute				

KEGG symbol	KEGG ID	KEGG product	NCBI locus tag of *M. soudanensis* NC18^T^	NCBI locus tag of *M. soudanensis* NC20
betA, CHDH	K00108	choline dehydrogenase	HQ775_RS19640, HQ775_RS24060, HQ775_RS25045, HQ775_RS09210, HQ775_RS11640	HQ843_RS11100, HQ843_RS05820, HQ843_RS05705, HQ843_RS21490, HQ843_RS19075, HQ843_RS13905, HQ843_RS03380
betB, gbsA	K00130	betaine-aldehyde dehydrogenase	HQ775_RS24935	HQ843_RS05815
CMO	K00499	choline monooxygenase	HQ775_RS19740	HQ843_RS11000
dctM	K11690	C4-dicarboxylate transporter, DctM subunit	HQ775_RS17665, HQ775_RS17705, HQ775_RS18830, HQ775_RS19705, HQ775_RS00200, HQ775_RS00660, HQ775_RS01695, HQ775_RS02905, HQ775_RS03120, HQ775_RS04820, HQ775_RS29050	HQ843_RS13070, HQ843_RS13030, HQ843_RS11910, HQ843_RS11035, HQ843_RS03525, HQ843_RS03440, HQ843_RS02405, HQ843_RS25865, HQ843_RS27255
dctP	K11688	C4-dicarboxylate-binding protein DctP	HQ775_RS04165	HQ843_RS26515
dctQ	K11689	C4-dicarboxylate transporter, DctQ subunit	HQ775_RS17760	HQ843_RS12975
ectC	K06720	L-ectoine synthase	HQ775_RS28260	HQ843_RS26775
kamA	K01843	lysine 2,3-aminomutase	HQ775_RS19895	HQ843_RS10845
opuBD	K05846	osmoprotectant transport system permease protein	HQ775_RS25205, HQ775_RS25215	HQ843_RS05545, HQ843_RS05535
opuC	K05845	osmoprotectant transport system substrate-binding protein	HQ775_RS25200, HQ775_RS27895	HQ843_RS05550, HQ843_RS28020
opuD, betL	K05020	glycine betaine transporter	HQ775_RS27890	HQ843_RS28025
pepQ	K01271	Xaa-Pro dipeptidase	HQ775_RS07200	HQ843_RS23495
proV	K02000	glycine betaine transport system ATP-binding protein	HQ775_RS10910	HQ843_RS09775, HQ843_RS05790, HQ843_RS19795
proW	K02001	glycine betaine transport system permease protein	HQ775_RS24955, HQ775_RS10905	HQ843_RS05795, HQ843_RS19800
proX	K02002	glycine betaine transport system substrate-binding protein	HQ775_RS10900	HQ843_RS19805
TC.BCT	K03451	betaine/carnitine transporter, BCCT family	HQ775_RS23930	HQ843_RS06820
yiaN	K21393	TRAP-type transport system large permease protein	HQ775_RS17765, HQ775_RS19690	HQ843_RS16050, HQ843_RS12970
kch, trkA, mthK, pch	K10716	voltage-gated potassium channel	HQ775_RS12215	HQ843_RS18505
kdpA	K01546	potassium-transporting ATPase potassium-binding subunit	HQ775_RS21590	HQ843_RS09155
kdpB	K01547	potassium-transporting ATPase ATP-binding subunit	HQ775_RS21595	HQ843_RS09150
kdpC	K01548	potassium-transporting ATPase KdpC subunit	HQ775_RS21585	HQ843_RS09160
kdpF	K01545	potassium-transporting ATPase subunit F	HQ775_RS21605	HQ843_RS09140
kup	K03549	KUP system potassium uptake protein	HQ775_RS10475	HQ843_RS20230
trkA, ktrA, ktrC	K03499	trk/ktr system potassium uptake protein	HQ775_RS12680	HQ843_RS18040
trkH, trkG, ktrB, ktrD	K03498	trk/ktr system potassium uptake protein	HQ775_RS15215	HQ843_RS15520
