# Supplementary material for: Genomic Analysis of Halotolerant Bacterial Strains Martelella soudanensis NC18T and NC20
Source: J Microbiol Biotechnol. 2022 Oct 13;32(11):1427–34. doi: 10.4014/jmb.2208.08011 (PMC9720073; doi:10.4014/jmb.2208.08011)
Supplement: Supplementary file 1 [file jmb-32-11-1427-supple.pdf]

J. Microbiol. Biotechnol.
https://doi.org/10.4014/jmb.2208.08011

Supplementary Tables

Genomic analysis of the halotolerant bacterial strains *Martelella soudanensis* NC18^T and NC20

Jung-Yun Lee[1, 2] and Dong-Hun Kim[1*]

[1]Groundwater Environment Research Center, Korea Institute of Geoscience and Mineral Resources, Daejeon 34132, Republic of Korea

[2]Department of Biological Science and Biotechnology, Microbiology & Biotechnology, Chungbuk National University, Cheongju 28644, Republic of Korea

Table S1. Functional categories information on the *Martelella soudanensis* NC18^T and NC20 genome based on KEGG database.

**KEGG functional categories**		**Number of genes**	
		**NC18^T**	**NC20**
**Metabolism**	Overview	1896	1891
	Carbohydrate metabolism	387	384
	Amino acid metabolism	309	309
	Energy metabolism	153	153
	Metabolism of cofactors and vitamins	153	153
	Xenobiotics biodegradation and metabolism	148	148
	Nucleotide metabolism	101	101
	Metabolism of other amino acids	79	79
	Glycan biosynthesis and metabolism	76	76
	Lipid metabolism	59	60
	Biosynthesis of other secondary metabolites	47	46
	Metabolism of terpenoids and polyketides	42	42
**Environmental information processing**	Membrane transport	185	185
	Signal transduction	117	116
	Signaling molecules and interaction	1	1
**Genetic information processing**	Translation	82	82
	Replication and repair	73	73
	Folding, sorting and degradation	42	42
	Transcription	6	6
**Human diseases**	Neurodegenerative disease	45	45
	Drug resistance: antimicrobial	33	33
	Cancer: overview	31	30
	Cardiovascular disease	20	20
	Infectious disease: bacterial	20	19
	Endocrine and metabolic disease	17	17
	Infectious disease: viral	12	12
	Drug resistance: antineoplastic	8	8
	Infectious disease: parasitic	7	7
	Cancer: specific types	6	6
	Immune disease	2	2
	Substance dependence	1	1
**Cellular processes**	Cellular community - prokaryotes	93	94
	Cell growth and death	33	33
	Transport and catabolism	19	19
	Cell motility	9	9
	Cellular community - eukaryotes	3	3
**Organismal systems**	Endocrine system	31	30
	Environmental adaptation	16	16
	Nervous system	15	15
	Digestive system	13	13
	Aging	12	12
	Immune system	10	10
	Circulatory system	5	5
	Sensory system	3	3
	Development and regeneration	2	2
	Excretory system	2	2
**Total number of genes**		4424	4413

Table S2. Functional categories information on the *Martelella soudanensis* NC18^T and NC20 genome based on COG database.

**COG functional categories**		**Number of genes**	
		**NC18^T**	**NC20**
**Metabolism**	Amino acid transport and metabolism (E)	526	537
	Carbohydrate transport and metabolism (G)	524	534
	Inorganic ion transport and metabolism (P)	451	458
	Energy production and conversion (C)	306	310
	Lipid transport and metabolism (I)	169	172
	Coenzyme transport and metabolism (H)	119	120
	Secondary metabolites biosynthesis, transport, and catabolism (Q)	118	118
	Nucleotide transport and metabolism (F)	99	102
**Information storage and processing**	Transcription (K)	496	500
	Replication, recombination, and repair (L)	302	303
	Translation, ribosomal structure, and biogenesis (J)	173	173
	Chromatin structure and dynamics (B)	1	1
**Cellular processes and signaling**	Cell wall/membrane/envelope biogenesis (M)	199	200
	Post-translational modification, protein turnover (O)	148	148
	Signal transduction mechanisms (T)	134	135
	Defense mechanisms (V)	89	90
	Intracellular trafficking, secretion, and vesicular transport (U)	59	59
	Cell cycle control, cell division, chromosome partitioning (D)	22	22
	Cell motility (N)	2	2
**Pooly chracterized**	Function unknown (S)	1530	1547
**Total number of genes**		5467	5531

Table S3. Functional categories information on the *Martelella soudanensis* NC18^T and NC20 genome based on SEED database.

SEED functional categories	Number of genes	
	**NC18^T**	**NC20**
Amino Acids and Derivatives	411	404
Carbohydrates	401	395
Protein Metabolism	205	204
Cofactors, Vitamins, Prosthetic Groups, Pigments	184	181
Membrane Transport	160	156
Fatty Acids, Lipids, and Isoprenoids	154	116
Nucleosides and Nucleotides	119	108
Respiration	97	97
Metabolism of Aromatic Compounds	95	93
Stress Response	94	91
DNA Metabolism	64	64
Virulence, Disease and Defense	58	58
RNA Metabolism	49	49
Nitrogen Metabolism	36	36
Cell Wall and Capsule	35	35
Miscellaneous	35	35
Regulation and Cell signaling	29	29
Phosphorus Metabolism	29	29
Iron acquisition and metabolism	20	20
Phages, Prophages, Transposable elements, Plasmids	15	15
Sulfur Metabolism	10	10
Potassium metabolism	9	9
Secondary Metabolism	8	8
Motility and Chemotaxis	6	5
Dormancy and Sporulation	1	1
**Total number of genes**	2324	2248

Table S4. Gene information in salt tolerance mechanism identified on the *Martelella soudanensis* NC18^T complete genome based on KEGG database.

Function	Type	RefSeq accession number	NCBI locus tag	KEGG product	Length (aa)	KEGG	
						Symbol	ID
Salt tolerance	Compatible solute	WP_180898255.1	HQ775_RS19640	choline dehydrogenase	513	betA, CHDH	K00108
		WP_180899248.1	HQ775_RS24060	choline dehydrogenase	410	betA, CHDH	K00108
		WP_180899424.1	HQ775_RS25045	choline dehydrogenase	526	betA, CHDH	K00108
		WP_180901279.1	HQ775_RS09210	choline dehydrogenase	538	betA, CHDH	K00108
		WP_180901686.1	HQ775_RS11640	choline dehydrogenase	537	betA, CHDH	K00108
		WP_180899404.1	HQ775_RS24935	betaine-aldehyde dehydrogenase	487	betB, gbsA	K00130
		WP_180898274.1	HQ775_RS19740	choline monooxygenase	376	CMO	K00499
		WP_180897904.1	HQ775_RS17665	C4-dicarboxylate transporter, DctM subunit	430	dctM	K11690
		WP_180897910.1	HQ775_RS17705	C4-dicarboxylate transporter, DctM subunit	451	dctM	K11690
		WP_180898115.1	HQ775_RS18830	C4-dicarboxylate transporter, DctM subunit	422	dctM	K11690
		WP_180898267.1	HQ775_RS19705	C4-dicarboxylate transporter, DctM subunit	423	dctM	K11690
		WP_180899733.1	HQ775_RS00200	C4-dicarboxylate transporter, DctM subunit	432	dctM	K11690
		WP_180899821.1	HQ775_RS00660	C4-dicarboxylate transporter, DctM subunit	438	dctM	K11690
		WP_180899989.1	HQ775_RS01695	C4-dicarboxylate transporter, DctM subunit	434	dctM	K11690
		WP_180900200.1	HQ775_RS02905	C4-dicarboxylate transporter, DctM subunit	427	dctM	K11690
		WP_180900235.1	HQ775_RS03120	C4-dicarboxylate transporter, DctM subunit	403	dctM	K11690
		WP_180900514.1	HQ775_RS04820	C4-dicarboxylate transporter, DctM subunit	437	dctM	K11690
		WP_180902630.1	HQ775_RS29050	C4-dicarboxylate transporter, DctM subunit	430	dctM	K11690
		WP_180900402.1	HQ775_RS04165	C4-dicarboxylate-binding protein DctP	336	dctP	K11688
		WP_180897921.1	HQ775_RS17760	C4-dicarboxylate transporter, DctQ subunit	174	dctQ	K11689
		WP_180902707.1	HQ775_RS28260	L-ectoine synthase	122	ectC	K06720
		WP_180898300.1	HQ775_RS19895	lysine 2,3-aminomutase	352	kamA	K01843
		WP_180899455.1	HQ775_RS25205	osmoprotectant transport system permease protein	397	opuBD	K05846
		WP_180899457.1	HQ775_RS25215	osmoprotectant transport system permease protein	246	opuBD	K05846

		WP_180899454.1	HQ775_RS25200	osmoprotectant transport system substrate-binding protein	305	opuC	K05845
		WP_180902441.1	HQ775_RS27895	osmoprotectant transport system substrate-binding protein	613	opuC	K05845
		WP_180902440.1	HQ775_RS27890	glycine betaine transporter	505	opuD, betL	K05020
		WP_180900932.1	HQ775_RS07200	Xaa-Pro dipeptidase	383	pepQ	K01271
		WP_180901565.1	HQ775_RS10910	glycine betaine/proline transport system ATP-binding protein	414	proV	K02000
		WP_180899407.1	HQ775_RS24955	glycine betaine/proline transport system permease protein	278	proW	K02001
		WP_180901564.1	HQ775_RS10905	glycine betaine/proline transport system permease protein	303	proW	K02001
		WP_180901563.1	HQ775_RS10900	glycine betaine/proline transport system substrate-binding protein	335	proX	K02002
		WP_180899223.1	HQ775_RS23930	betaine/carnitine transporter, BCCT family	537	TC.BCT	K03451
		WP_180897922.1	HQ775_RS17765	TRAP-type transport system large permease protein	431	yiaN	K21393
		WP_180898264.1	HQ775_RS19690	TRAP-type transport system large permease protein	647	yiaN	K21393
	**Salt-in**	WP_180902135.1	HQ775_RS12215	voltage-gated potassium channel	352	kch, trkA, mthK, pch	K10716
		WP_180898686.1	HQ775_RS21590	potassium-transporting ATPase potassium-binding subunit	557	kdpA	K01546
		WP_180898688.1	HQ775_RS21595	potassium-transporting ATPase ATP-binding subunit	690	kdpB	K01547
		WP_180898685.1	HQ775_RS21585	potassium-transporting ATPase KdpC subunit	184	kdpC	K01548
		WP_180898692.1	HQ775_RS21605	potassium-transporting ATPase subunit F	29	kdpF	K01545
		WP_210275347.1	HQ775_RS10475	KUP system potassium uptake protein	611	kup	K03549
		WP_180901854.1	HQ775_RS12680	trk/ktr system potassium uptake protein	458	trkA, ktrA, ktrC	K03499
		WP_180897475.1	HQ775_RS15215	trk/ktr system potassium uptake protein	484	trkH, trkG, ktrB, ktrD	K03498

Table S5. Gene information in salt tolerance mechanism identified on the *Martelella soudanensis* NC20 complete genome based on KEGG database.

Function	Type	RefSeq accession number	NCBI locus tag	KEGG product	Length (aa)	KEGG	
						Symbol	ID
Salt tolerance	Compatible solute	WP_180898255.1	HQ843_RS11100	choline dehydrogenase	513	betA, CHDH	K00108
		WP_180899403.1	HQ843_RS05820	choline dehydrogenase	559	betA, CHDH	K00108
		WP_180899424.1	HQ843_RS05705	choline dehydrogenase	526	betA, CHDH	K00108
		WP_180901279.1	HQ843_RS21490	choline dehydrogenase	538	betA, CHDH	K00108
		WP_180901686.1	HQ843_RS19075	choline dehydrogenase	537	betA, CHDH	K00108
		WP_180903226.1	HQ843_RS13905	choline dehydrogenase	545	betA, CHDH	K00108
		WP_180903399.1	HQ843_RS03380	choline dehydrogenase	537	betA, CHDH	K00108
		WP_180899404.1	HQ843_RS05815	betaine-aldehyde dehydrogenase	487	betB, gbsA	K00130
		WP_180898274.1	HQ843_RS11000	choline monooxygenase	376	CMO	K00499
		WP_180898274.1	HQ843_RS11000	choline monooxygenase	376	CMO	K00499
		WP_180897904.1	HQ843_RS13070	C4-dicarboxylate transporter, DctM subunit	430	dctM	K11690
		WP_180897910.1	HQ843_RS13030	C4-dicarboxylate transporter, DctM subunit	451	dctM	K11690
		WP_180898115.1	HQ843_RS11910	C4-dicarboxylate transporter, DctM subunit	422	dctM	K11690
		WP_180898267.1	HQ843_RS11035	C4-dicarboxylate transporter, DctM subunit	423	dctM	K11690
		WP_180899804.1	HQ843_RS03525	C4-dicarboxylate transporter, DctM subunit	429	dctM	K11690
		WP_180899821.1	HQ843_RS03440	C4-dicarboxylate transporter, DctM subunit	438	dctM	K11690
		WP_180899989.1	HQ843_RS02405	C4-dicarboxylate transporter, DctM subunit	434	dctM	K11690
		WP_180900514.1	HQ843_RS25865	C4-dicarboxylate transporter, DctM subunit	437	dctM	K11690
		WP_180902630.1	HQ843_RS27255	C4-dicarboxylate transporter, DctM subunit	430	dctM	K11690
		WP_180900402.1	HQ843_RS26515	C4-dicarboxylate-binding protein DctP	336	dctP	K11688
		WP_180897921.1	HQ843_RS12975	C4-dicarboxylate transporter, DctQ subunit	174	dctQ	K11689
		WP_180902707.1	HQ843_RS26775	L-ectoine synthase	122	ectC	K06720
		WP_180898300.1	HQ843_RS10845	lysine 2,3-aminomutase	352	kamA	K01843

		WP_180899455.1	HQ843_RS05545	osmoprotectant transport system permease protein	397	opuBD	K05846
		WP_180899457.1	HQ843_RS05535	osmoprotectant transport system permease protein	246	opuBD	K05846
		WP_180899454.1	HQ843_RS05550	osmoprotectant transport system substrate-binding protein	305	opuC	K05845
		WP_180902441.1	HQ843_RS28020	osmoprotectant transport system substrate-binding protein	613	opuC	K05845
		WP_180902440.1	HQ843_RS28025	glycine betaine transporter	505	opuD, betL	K05020
		WP_180900932.1	HQ843_RS23495	Xaa-Pro dipeptidase	383	pepQ	K01271
		WP_180898485.1	HQ843_RS09775	glycine betaine/proline transport system ATP-binding protein	352	proV	K02000
		WP_180899408.1	HQ843_RS05790	glycine betaine/proline transport system ATP-binding protein	346	proV	K02000
		WP_180901565.1	HQ843_RS19795	glycine betaine/proline transport system ATP-binding protein	414	proV	K02000
		WP_180899407.1	HQ843_RS05795	glycine betaine/proline transport system permease protein	278	proW	K02001
		WP_180901564.1	HQ843_RS19800	glycine betaine/proline transport system permease protein	303	proW	K02001
		WP_180901563.1	HQ843_RS19805	glycine betaine/proline transport system substrate-binding protein	335	proX	K02002
		WP_180899223.1	HQ843_RS06820	betaine/carnitine transporter, BCCT family	537	TC.BCT	K03451
		WP_180897380.1	HQ843_RS16050	TRAP-type transport system large permease protein	431	yiaN	K21393
		WP_180897922.1	HQ843_RS12970	TRAP-type transport system large permease protein	431	yiaN	K21393
	**Salt-in**	WP_180902135.1	HQ843_RS18505	voltage-gated potassium channel	352	kch, trkA, mthK, pch	K10716
		WP_180898686.1	HQ843_RS09155	potassium-transporting ATPase potassium-binding subunit	557	kdpA	K01546
		WP_180898688.1	HQ843_RS09150	potassium-transporting ATPase ATP-binding subunit	690	kdpB	K01547
		WP_180898685.1	HQ843_RS09160	potassium-transporting ATPase KdpC subunit	184	kdpC	K01548
		WP_180898692.1	HQ843_RS09140	potassium-transporting ATPase subunit F	29	kdpF	K01545
		WP_210275347.1	HQ843_RS20230	KUP system potassium uptake protein	611	kup	K03549
		WP_180901854.1	HQ843_RS18040	trk/ktr system potassium uptake protein	458	trkA, ktrA, ktrC	K03499
		WP_180897475.1	HQ843_RS15520	trk/ktr system potassium uptake protein	484	trkH, trkG, ktrB, ktrD	K03498
